# Selective Wnt/β-Catenin Pathway Activation Concomitant With Sustained Overexpression of miR-21 is Responsible for Aristolochic Acid-Induced AKI-to-CKD Transition

**DOI:** 10.3389/fphar.2021.667282

**Published:** 2021-05-28

**Authors:** Qing Kuang, Sheng Wu, Ning Xue, Xiaoyan Wang, Xiaoqianq Ding, Yi Fang

**Affiliations:** ^1^ Department of Nephrology, Zhongshan Hospital, Fudan University, Shanghai, China; ^2^ Department of Nephrology, Suzhou Dushuhu Public Hospital, Suzhou, China; ^3^ Shanghai Medical Center of Kidney, Shanghai, China; ^4^ Shanghai Institute of Kidney and Dialysis, Shanghai, China; ^5^ Shanghai Key Laboratory of Kidney and Blood Purification, Shanghai, China

**Keywords:** Wnt, β-catenin, miR-21, AKI-CKD transition, aristolochic acid

## Abstract

Acute kidney injury (AKI) is increasingly recognized as a cumulative risk factor for chronic kidney disease (CKD) progression. However, the underlying mechanisms remain unclear. Using an aristolochic acid (AA)-induced mouse model of AKI-to-CKD transition, we found that the development of tubulointerstitial fibrosis following AKI was accompanied with a strong activation of miR-21 and canonical Wnt signaling, whereas inhibition of miR-21 or selective silencing of Wnt ligands partially attenuated AKI-to-CKD transition. To explore the interaction between miR-21 and Wnt/β-catenin signaling, we examined the effects of genetic absence or pharmacologic inhibition of miR-21 on Wnt/β-catenin pathway expression. In miR-21^−/−^ mice and in wild-type mice treated with anti-miR21 oligos, Wnt1 and Wnt4 canonical signaling in the renal tissue was significantly reduced, with partial reversal of renal interstitial fibrosis. Although the renal abundance of miR-21 remained unchanged after inhibition or activation of Wnt/β-catenin signaling, early intervention with ICG-001, a β-catenin inhibitor, significantly attenuated renal interstitial fibrosis. Moreover, early (within 24 h), but not late β-catenin inhibition after AA administration attenuated AA-induced apoptosis and inflammation. In conclusion, inhibition of miR-21 or β-catenin signaling may be an effective approach to prevent AKI-to-CKD progression.

## Introduction

Acute kidney injury (AKI) is a common complication among hospitalized patients associated with high mortality, increased in-hospital costs, and excessive length of stay (Fang et al., 2015). Recent epidemiological studies highlighted that even patients who survive a short-term insult of AKI have a poor prognosis (Lo et al., 2009; Thakar et al., 2011). Emerging studies indicate that the duration, severity, and frequency of AKI are related to the development and progression of chronic kidney disease (CKD) and even end-stage renal disease (ESRD) in survivors of AKI, suggesting that AKI episodes independently increase the risk of subsequent chronic, progressive kidney dysfunction (Amdur et al., 2009; Brown et al., 2010). However, the mechanisms driving AKI-to-CKD transition remain unclear, and no effective interventions or treatments are available (Chawla and Kimmel, 2012; Coca et al., 2012).

MicroRNAs (miRNAs or miRs) are a class of endogenous, small, noncoding RNA molecules of approximately 22 nucleotides in length that have complex and diverse physiological functions (Ambros, 2004). MiR-21, one of the most widely studied miRNAs, is importantly involved in tissue repair, proliferation, apoptosis, and diverse pathophysiological processes secondary to AKI (Xu et al., 2012; Lai et al., 2015; Jia et al., 2017). Animal studies have revealed that miR-21 abundance was substantially increased in both the renal tubulointerstitium and glomeruli when fibrosis occurred, which indicated that miR-21 might be involved in regulating post-injury fibrosis of the kidney (Zhong et al., 2011; Wang et al., 2012).

The Wnt family comprises a group of highly conserved, secreted signaling glycoproteins that play critical roles in stem cell differentiation, kidney development, and tissue homeostasis. Despite being relatively silent in renal tissue under normal circumstances, Wnt/β-catenin signaling is activated after injury (Angers and Moon, 2009; Clevers and Nusse, 2012). Mounting studies (Terada et al., 2003; Wang et al., 2009; Zhou et al., 2012) show that the upregulation of Wnt/β-catenin signaling is advantageous for renal tubular recovery; however, sustained overexpression of this pathway is pernicious and appears to be a common pathologic pathway in the pathogenesis of fibrotic events, resulting in irreversible activation of the renin-angiotensin-aldosterone system, inflammatory response, and extracellular matrix deposition (He et al., 2009; Hao et al., 2011; Maarouf et al., 2016; Xiao et al., 2016). Thus, the duration of Wnt/β-catenin signaling activation may be essential for the development of CKD.

Considering the similar profiles of miR-21 and Wnt/β-catenin signaling throughout AKI-to-CKD transition, it is rational to hypothesize that there exists a relation between miR-21 and Wnt/β-catenin signaling. Therefore, we characterized the renal profiles of miR-21/Wnt signaling at different time points after aristolochic acid (AA)-induced acute nephrotoxic insult, which represents a suitable model of AKI-to-CKD. In addition, we investigated whether the abundance of miR-21 or the expression of Wnt/β-catenin signaling determines the fate of tubular epithelial cells, as well as the interaction between miR-21 and Wnt/β-catenin signaling.

## Materials and Methods

### Mouse Model Establishment and Experimental Setup

All experiments were performed using male C57BL/6 mice, which were obtained from the Animal Center of Fudan University, Shanghai, China. MiR-21 knockout mice were generated at the Shanghai Model Organisms Center as described previously (Jia et al., 2017). Adult mice (8- to 10-week-old; 20–25 g) used were housed in temperature- and humidity-controlled cages, with free access to water and food, under a 12 h light/dark cycle. All animal experiments were approved by the Institutional Animal Care and Use Committee of Fudan University and were performed in accordance with the National Institutes of Health Guide for the Care and Use of Laboratory Animals.

Aristolochic acid I sodium salt (Sigma) was diluted in saline (0.5 mg/ml) and was administered to mice by a single intraperitoneal injection at a bolus dose of 10 mg/kg to establish an AKI-to-CKD model. The mice were sacrificed at days 1, 3, 7 14, or 28 after injected with AA.

To study the role of Wnt/β-catenin, two experiments were performed. In the first experiment, ICG-001-phosphate (5 mg/kg) was dissolved in dimethyl sulfoxide (DMSO) at a concentration of 4 mg/dl and was administered daily to mice via intraperitoneal injection for consecutive 7 days, starting 24 h after AA delivery or 7 days after AA delivery. In the second experiment, animals were injected intraperitoneally of LiCl (Sigma, 20 mg/kg/d, diluted in normal saline as 10 mg/ml), starting from 24 h post AA treatment, for 7 consecutive days. Mice were sacrificed and kidney tissues were collected for indicated analyses 14 and 28 days post AA administration, or at indicated time points.

### MiRNA Knockdown *In Vivo*

For testing the effects of pharmacologic inhibition of miR-21, locked nucleic acid- (LNA-) modified anti-miR21 oligonucleotides or anti-scramble (Exiqon) was dissolved in saline (5 mg/ml) and administered via the tail vein (10 mg/kg) within 60 min prior to AA delivery (Xu et al., 2012). Additional injections (the same dose) were given on the 5th and 10th day after AA-I dosing. All the experiments were replicated at least twice.

### MiRNA Genetic Knockout *In Vivo*

MiR-21 knockout mice were generated at the Shanghai Model Organisms Center, Inc. (Jia et al., 2017). In brief, an frt-PGK-Neo-frt cassette followed by 5′ loxP site was introduced upstream of *miR-21* gene and 3′ loxP site was inserted downstream of *miR-21* gene. The targeting vector was introduced into SCR012 ES cells. Targeted ES clones were microinjected into ICR embryos and transferred into pseudopregnant ICR females. After bred with C57BL/6 mice, these heterozygous mice were mated with ACTFLPe transgenic mice to remove Neo cassette. After backcrossed with C57BL/6, heterozygous mice (*miR-21* ^flox/+^) were mated with *EIIA-Cre* transgenic mice to obtain miR-21 knockout mice (*miR-21* ^+/−^) which were mated to generate homozygous mutants (*miR-21* ^−/−^). Then, homozygous mutant mice mated with each other to obtain more *miR-21* ^−/−^ mice. *miR-21* knockout mice were identified by the following primer pairs: Forward, 5′ -CAG​AAT​TGC​CCA​GGC​TTT​TA -3′; Reverse, 5′- AAT​CCA​TGA​GGC​AAG​GTG​AC -3′.

### Cell Culture and Treatments

HK-2 human proximal tubular epithelial cells were cultured in DMEM/F12 supplemented with 10% fetal bovine serum in an incubator with 5% CO_2_ at 37°C.

For AA treatment, the cells were cultured in 6-well plates and randomly assigned to experimental groups. Cells were cultured in complete medium with AA (0, 1, 2, 5, 10 μg/ml) for 24, 48, and 72 h, for indicated *in-vitro* experiments. In order to suppress *in-vitro* miR-21 levels when HK-2 cells reached 60–70% confluency, 50 nM anti-miR21 or 50 nM anti-scramble (Exiqon) was respectively transfected into cells 6 h prior to AA treatment (2 μg/ml) by using Lipofectamine 2000 (Invitrogen) according to the manufacturer’s instructions, following which the medium was changed and cells were harvested 48 h after AA treatment. In order to inhibit β-catenin, HK-2 cells were exposed to different concentrations of DKK-1 (50, 100, 150 ng/ml) diluted in DMSO for 1 h, prior to 48 h AA exposure (2 μg/ml). All the experiments were replicated at least twice.

Interfering RNAs (siRNAs) targeting Wnt1, Wnt4 and NF-κB subunit p65 (RelA) or an RNA duplex with random sequence as negative control (NC) were transfected into HK2 cells at 50 nM 6 h (for Wnt1/Wnt4 siRNA) or at 100 nM12 h (for NF-κB p65 siRNA) prior to AA treatment (2 μg/ml) by using Lipofectamine 2000 (Invitrogen) according to the manufacturer’s instructions. The RNA transfection efficiency was 65–80% for at least 48 h. RNA oligoribonucleotides were obtained from GenePharma (Shanghai, China) and Ribo Life Science (Suzhou, China) and the sequences are listed in Table 1.

**TABLE 1:** siRNA sequences targeting Wnt1 and Wnt4.

Target	Sequence
Wnt1 (human)	Sense: 5′-CUC​GUC​UAC​UUC​GAG​AAA​UTT-3′
	Antisense: 5′-AUU​UCU​CGA​AGU​AGA​CGA​GTT-3′
Wnt4 (human)	Sense: 5′-GCU​GCA​GAG​AUC​AAA​GAA​ATT-3′
	Antisense: 5′-UUU​CUU​UGA​UCU​CUG​CAG​CTT-3′
RelA (human)	Sense: 5′-GCA​UCC​AGA​CCA​ACA​ACA​A -3′
	Antisense: 5′-UUG​UUG​UUG​GUC​UGG​AUG​C -3′
Negative control (human)	Sense: 5′-UUC​UCC​GAA​CGU​GUC​ACG​UTT-3′
	Antisense: 5′-ACG​UGA​CAC​GUU​CGG​AGA​ATT-3′

### Immunohistochemistry

Changes in renal morphology were examined in paraffin-embedded tissue sections (4 μm) stained with hematoxylin. Immunohistochemical staining was performed as previously described (Xu et al., 2012). The following primary antibodies were used: anti-Wnt1 (1:100, Abcam), anti-Wnt4 (1:150, Santa Cruz Biotechnology), anti-c-caspase3 (1:500, Cell Signaling Technology), anti-F4/80 (1:200, Abcam). The secondary antibody was horseradish peroxidase-conjugated anti-mouse IgG (1:1,000; Jackson ImmnoResearch), which was used according to the manufacturer’s instructions. The peroxidase was visualized with diaminobenzidine. To determine the number of F4/80-positive cells, 8–10 fields for each mouse were examined under a microscope (magnification, 200×), and the average number of positive cells was calculated.

### Western Blot Analysis

The relative protein abundances in kidneys and HK-2 cells were analyzed using western blotting, as described previously (Xu et al., 2012). The following primary antibodies were used: anti-α-SMA (1:1,000, Sigma), anti-E-cadherin (1:1,000, Cell Signaling Technology), anti-vimentin (1:1,000, Santa Cruz Biotechnology), anti-collagen I (1:1,000, Abcam), anti-collagen IV (1:1,000, Abcam), anti-Wnt1 (1:100, Abcam), anti-Wnt4 (1:150, Santa Cruz Biotechnology), anti-Wnt3 (1:500, Abcam), anti-Wnt2b (1:700, Abcam), anti-Wnt7a (1:300, Abcam), anti-β-catenin (1:1,000, Cell Signaling Technology), anti-Snail 1 (1:1,000, Santa Cruz Biotechnology), anti-PAI-1 (1:1,000, Santa Cruz Biotechnology), and anti-NF-κB p65 (1:1,000, Cell Signal). Horseradish peroxidase-conjugated secondary antibodies (anti-rabbit or anti-mouse or anti-goat IgG, 1:5,000, Jackson ImmunoResearch) were used according to the manufacturer’s instructions. Image analysis software (Image J, National Institutes of Health) was used to determine the gray value of all bands.

### Real-Time Reverse-Transcription PCR

Total RNA was isolated from kidney tissues and cells using TRIzol RNA Isolation System (Invitrogen). MiR-21 expression was quantified by RT-qPCR using Taqman chemistry (Applied Biosystems), as described previously, and was normalized to U6 small nuclear RNA expression (Jia et al., 2017). qRT-PCR was performed on an ABI PRISM 7000 Sequence Detection System (Applied Biosystems, Foster City, CA, United States). mRNA levels of *SOD2*, *PGC*-1α, and *Mpv17* were quantified using SYBR Green PCR Master Mix (Applied Biosystems), as previously described (Xiao et al., 2016), and were normalized to the level of 18S rRNA. The 2^–ΔΔCt^ method was used to determine relative changes in mRNA and miR-21 expression. Relative gene levels were expressed as ratios to the control.

### Histological Assessment

Kidneys were fixed in 10% formalin overnight, dehydrated with an ethanol gradient, cleared in xylene, embedded in paraffin, and cut into 4 μm-thick sections. Histopathological changes in the corticomedullary junction were assessed by H&E and Masson’s trichrome staining. Tissue damage was scored according to the following scale: no injury, “0”; mild (<25% staining) “1”; moderate (<50%) “2”; extensive (>50%) “3”; very severe (>75%) “4.” An average of 10 fields for each mouse were examined under a microscope at a magnification of 200× or 400× (Xu et al., 2012).

### Luciferase Assay

The 500 bp 3′UTR of human Wnt2b containing potential miR-21 binding site and its mutant were amplified by PCR and cloned into pMIR-REPORT vector, respectively. For luciferase reporter assays, cells were transiently transfected with pMIR-REPORT vectors together with indicated miR mimics using Lipofectamine 2000 for 24 h. Reporter activity was measured by the dual-luciferase assay-system (Promega). Renilla luciferase activity was used to normalize for transfection efficiency. The data were presented as fold change relative to the control group.

### Chromatin Immunoprecipitation Assay

Mouse renal tubular epithelial cells (mTECs) with indicated treatments were cross-linked with 1% formaldehyde for 10 min at 37°C and chromatin immunoprecipitation (ChIP) assay was performed using the Pierce Agarose ChIP Kit from Thermo (26156). Five micrograms of NF-κB p65 (Cell Signal) antibody was added for each assay and normal IgG were used as negative control. The miR-21 promoter region covering the potential NF-κB binding site (−900 ∼ −1,200 bp relative to its start codon in human miR-21 gene, −1 ∼ −300 bp in mouse miR-21 gene) was assayed for NF-κB binding by PCR using Premix Taq™ from Takara (RR900Q) and following primers. ATA​CTT​TTT​CCT​TCT​GTG​CTA​AGG​T (forward) and ACA​TGA​TAA​ACA​TGC​AAG​ACT​GTT​A (reverse) for human; CAG​AGG​ACA​CTA​GCA​AGA​AAG​GCT​T (forward) and GCC​ATG​CGA​TGT​CAC​GAC​CAC​GAC​A (reverse) for mouse. The PCR products were analyzed by 2% agarose electrophoresis.

### Serum Creatinine Assay

Blood was sampled from mouse eyes at the indicated times. Serum creatinine was detected by the improved Jaffe method using a Quantichrom™ creatinine Assay Kit (BioAssay Systems).

### Antioxidant Activity Assay

GSH-Px activity in the mouse kidney was detected using a glutathione peroxidase (GSH-PX) assay kit (colorimetric method; Nanjing Jiancheng Bioengineering Institute) per the manufacturer’s instructions. The change in absorbance at 412 nm during the conversion of GSH to oxidized glutathione was measured.

### Suspension Cytokine Array

The suspension cytokine arrays were used to verify the concentration of cytokines in mice samples. The mouse cytokines of IL-10, IL-13, Eotaxin, chemokine (C-X-C motif) ligand 1 (CXCl-1), monocyte chemotactic protein 1 (MCP-1), macrophage inflammatory protein-1a (MIP-1a), and macrophage inflammatory protein-1b (MIP-1b) were quantified using Mouse 23-plex Multi-Analyte Kit (Bio-Plex Suspension Array System; Bio-Rad, Hercules, CA, United States) according to the manufacturer’s instructions. In brief, 50 μL of sample was added to each well, and incubated with capture antibody-coupled beads in the dark at room temperature with shaking at 850 rpm for 2 h. After washing with 3 times, 50 μL biotinylated antibody was added and then incubated for 1 h. The captured cytokines were visualized with streptavidin-phycoerythrin (PE). Finally, the plate was read using a Bio-Plex MAGPIX Multiplex Reader (Bio-Rad, Hercules, CA, United States). Bio-Plex Manager 6.0 software was used for data acquisition and analysis.

### Statistical Analysis

The statistical software SPSS version 20.0 (SPSS Inc.) was used for statistical analysis. For continuous variables, data are presented as the mean ± SD. Multigroup differences were compared by one-way analysis of variance followed by Bonferroni’s post-hoc test. *p* < 0.05 was considered significant.

## Results

### MiR-21 is Sustained Activated After Aristolochic Acid Exposure *In Vivo* and *Vitro*

After a single intraperitoneal administration of aristolochic acid (10 mg/kg), the mice were sacrificed at different time points (days 1, 3, 7, 14, 28 after AA delivery), and a drug-related kidney injury model was successfully established. The mouse serum creatinine and the miR-21 level began to rise at 3 days post AA administration, peaked at day 14, and maintained a high level thereafter (Supplementary Figures S1A,B). Kidney histological examination revealed comparable results: severe renal tubular damage occurred on day 3, renal tubular atrophy along with interstitial fibrosis were observed on day 14, and more obvious on day 28 (Supplementary Figures S1C–E).

Next, we investigated the expression of miR-21 in cultured tubular epithelial (HK-2) cells treated with different doses of AA to delineate the mechanisms driving AKI-to-CKD progression. Even at the lowest doses of AA tested (1 or 2 μg/ml), miR-21 abundance in HK2 cells started to increase within 48 h after treatment, and then returned to baseline (Supplementary Figure S1F). Moreover, low-dose AA could induce cell phenotypic changes on account of the activation of some markers of epithelial-mesenchymal transition (EMT), such as α-smooth muscle actin (α-SMA), collagen I, and collagen IV. However, there was no change in the protein abundance of vimentin (Supplementary Figures S1G,H).

### Inhibition of miR⁃21 Partially Reverses Renal Tubulointerstitial Fibrosis Induced by Aristolochic Acid

**FIGURE 1:**
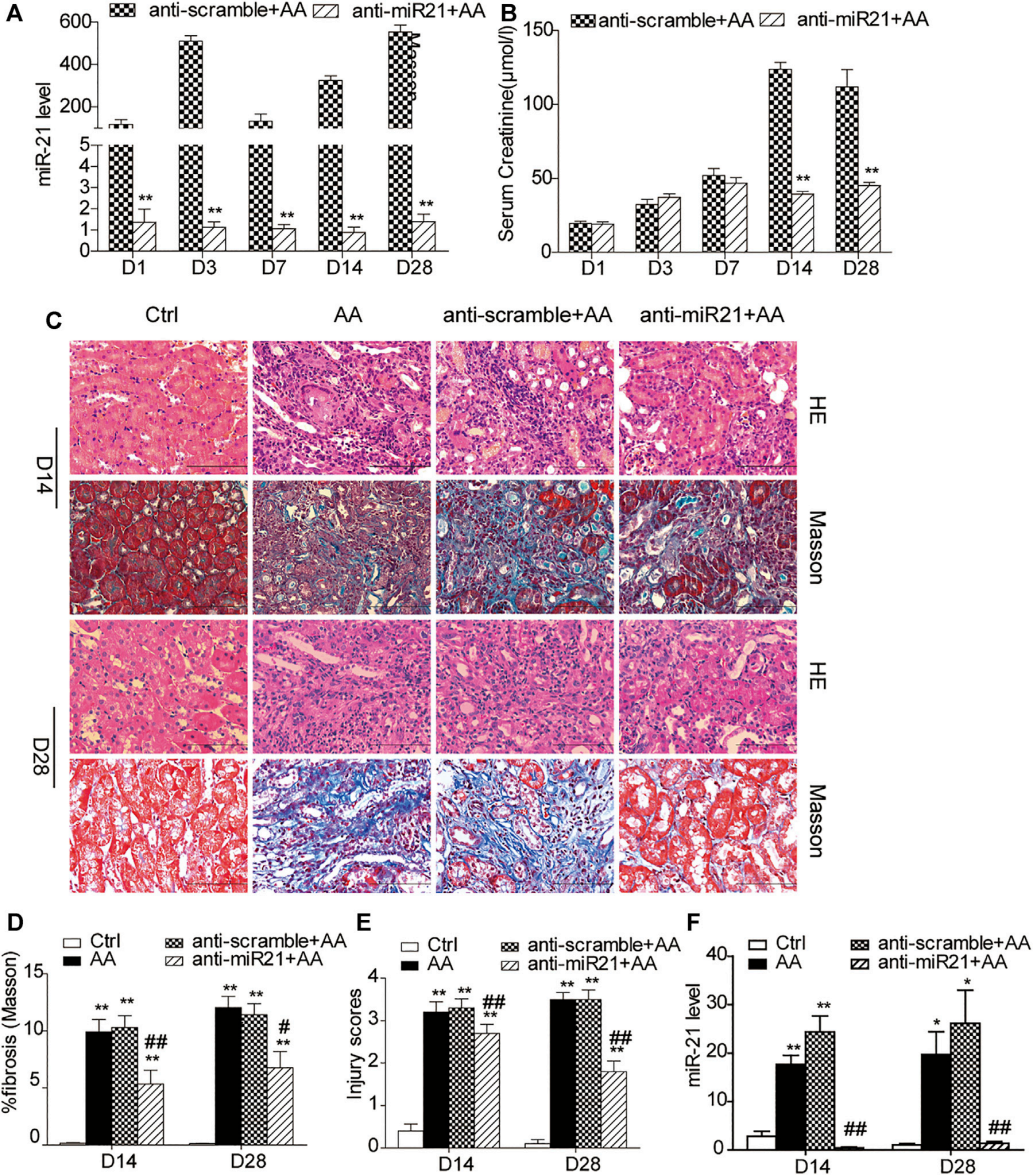
Inhibition of miR⁃21 partially reverses renal tubulointerstitial fibrosis induced by aristolochic acid. **(A,B)** RT-qPCR analysis of miR-21 abundance and serum levels of creatinine. Mice were sacrificed on days 1, 3, 7, 14, or 28 after aristolochic acid (AA) administration (10 mg/kg, i.p.). Locked nucleic acid- (LNA-) modified anti-miR21 oligonucleotides or anti-scramble was administered via the tail vein (10 mg/kg, 5 mg/ml) within 60 min prior to AA delivery. Additional injections (the same dose) were given on the 5^th^ and 10^th^ day after AA-I dosing. *p < 0.05 and **p < 0.01 vs. anti-scramble + AA groups, *n* = 6. **(C)** H&E and Masson’s trichrome staining of the corticomedullary junction (magnification, 400×, bar = 200 μm) on days 14 and 28 after AA delivery. **(D,E)** Histopathological assessment of H&E and Masson’s trichrome staining. Ten high-power fields for each mouse were captured, and then the average number in the high-power fields was calculated for quantification. **(F)** The miR-21 abundance. *p < 0.05 and **p < 0.01 vs. control groups (Ctrl). ^#^ *p* < 0.05 and ^##^ *p* < 0.01 vs. anti-scramble + AA groups, *n* = 6.

We knocked down miR-21 by using locked nucleic acid (LNA) modification technology. Compared with the anti-scramble + AA group at each time point, the abundance of miR-21 was significantly reduced in the anti-miR21 + AA group (Figure 1A). However, the decrease in serum creatinine level was more significant in the late stage of AA intervention (day 14 and day 28) (Figure 1B). Both H&E staining and Masson’s trichrome staining revealed a similar pattern to that of serum creatinine on day 14 and 28 (Figures 1C–E). The pathological changes in the anti-scramble + AA and AA groups were manifested as larger area of renal tubular necrosis, massive monocytes infiltration, and severe renal tubulointerstitial fibrosis (Figure 1C). And inhibition of miR-21 partially reduced kidney injury and renal fibrosis (Figures 1D–F).

### Wnt1/4-Mediated β-Catenin Pathways are Persistently Activated After Aristolochic Acid Exposure *In Vivo* and *Vitro*

**FIGURE 2:**
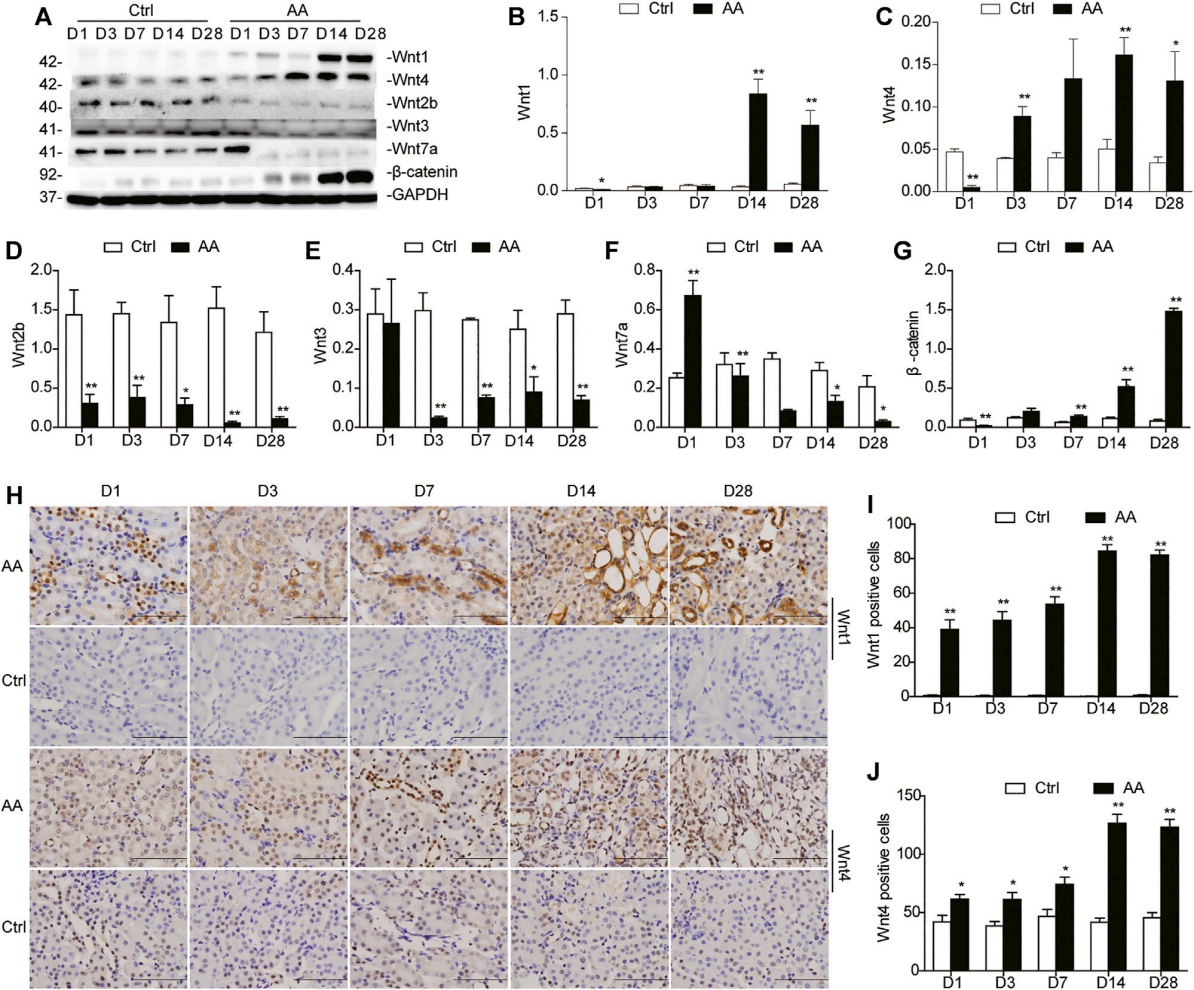
Sustained Wnt/β-catenin pathway activation secondary to AA induces AKI. **(A)** Western blot of representative Wnt ligands (Wnt1, Wnt2b, Wnt3, Wnt4, Wnt7a) and β-catenin in mouse kidney tissues. Mice were sacrificed on days 1, 3, 7, 14, or 28 after AA administration. **(B–G)** Quantitative western blot data. **(H)** Immunohistochemical staining of Wnt1 and Wnt4 in the kidneys (magnification, 400×, bar = 200 μm). **(I,J)** The number of Wnt1/Wnt4 positive cells in the mouse kidneys. Each mouse was calculated for quantification of 10 high-power fields. *p < 0.05 and **p < 0.01 vs. control groups (Ctrl), *n* = 6.

We first verified the dynamic expression patterns of miR-21 and Wnt/β-catenin signaling in mouse kidneys at different time points after high-dose AA (10 mg/kg) administration. The expression of Wnt ligands was evaluated on days 1, 3, 7, 14, and 28 after AA administration (Figure 2). The western blot analyses revealed that protein abundances of Wnt 1 and Wnt 4 displayed similar patterns to that of miR-21 (Figures 2A–C; Supplementary Figure S1B). Comparable results were obtained by immunohistochemical staining (Figures 2H–J). Furthermore, the two ligands were predominantly distributed in the renal tubule and interstitium, suggesting that they might be involved in the development of renal tubulointerstitial fibrosis. In contrast, other Wnt ligands, including Wnt 2b, Wnt 3, and Wnt 7a, exhibited in an opposite trend, reaching minimum expression values on day 28 (Figures 2A–F). Notably, western blotting also revealed that the renal β-catenin protein level gradually increased, lagging behind Wnt1 or Wnt4, peaking on day 28 (Figure 2G). Together, these data indicated that both miR-21 and Wnt/β-catenin were upregulated and likely involved in kidney fate after acute insult of AA exposure.

We also investigated the expression of Wnt1/4-mediated β-catenin pathways in HK-2 cells treated with different doses of AA. A trend similar to miR-21 was observed. The protein levels of Wnt1, Wnt4, and β-catenin in HK2 cells started to increase within 48 h after treatment, and then returned to baseline (Supplementary Figure S1G).

### Inhibition of miR-21 Attenuates AA-Induced Upregulation of Wnt1/4-Mediated β-Catenin Signaling *In Vivo* and *Vitro*

**FIGURE 3:**
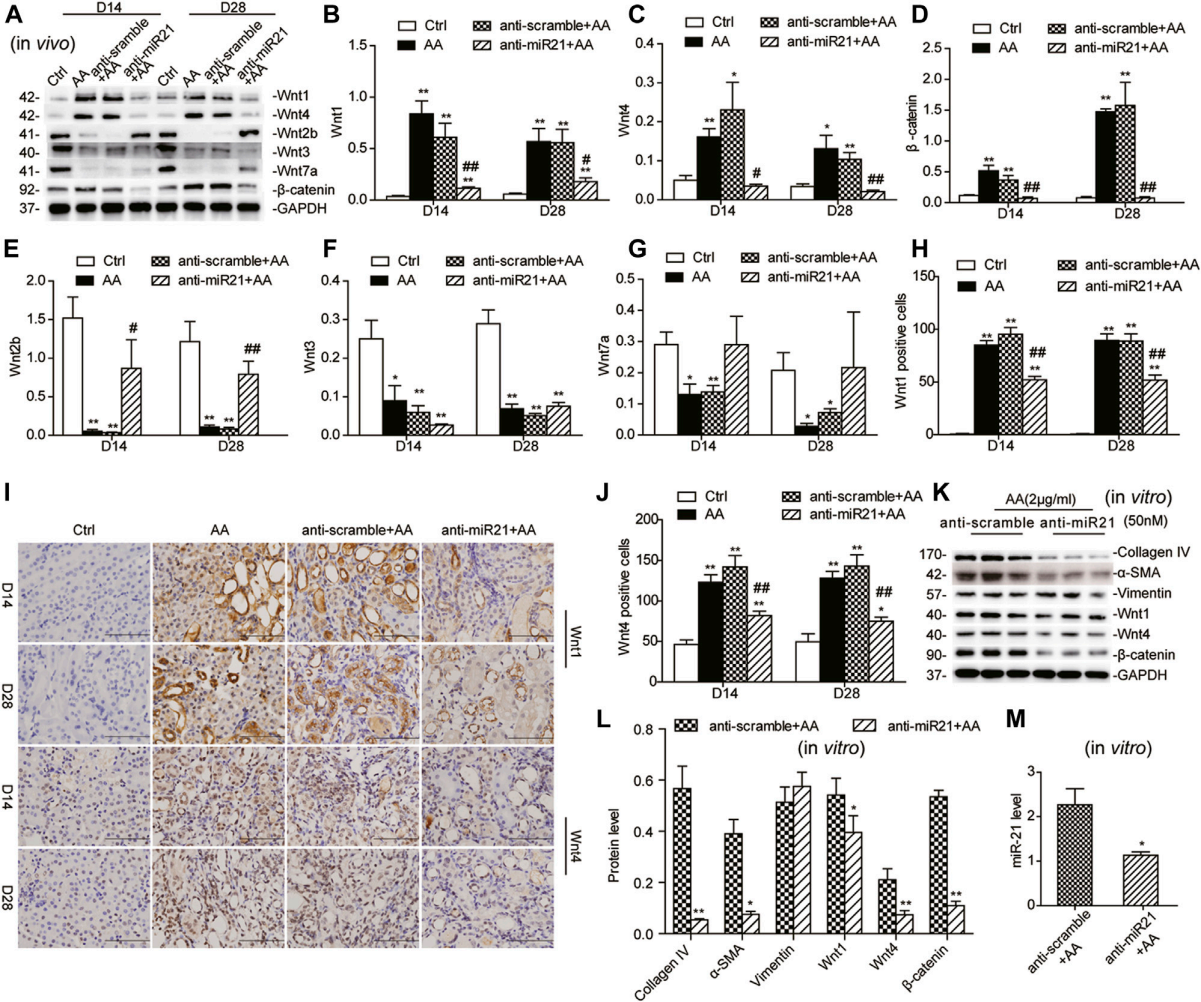
Inhibition of miR-21 selectively suppresses Wnt/β-catenin signaling *in vivo* and vitro. **(A–G)** Western blots and quantitative data of renal expression of Wnt1, Wnt2b, Wnt3, Wnt4, Wnt7a, and β-catenin on days 14 and 28 after AA delivery. LNA-modified anti-miR21 oligonucleotides or anti-scramble was administered to the mice via the tail vein (10 mg/kg, 5 mg/ml) within 60 min prior to AA delivery. Additional injections (the same dose) were given on the 5^th^ and 10^th^ day after AA-I dosing. **(H–J)** Immunohistochemical staining of Wnt1 and Wnt4 in the kidneys (magnification, 400×, bar = 200 μm) and the number of Wnt1/Wnt4 positive cells in the mouse kidneys. *p < 0.05, **p < 0.01 vs. controls. ^#^ *p* < 0.05, ^##^ *p* < 0.01 vs. anti-scrambled control groups, *n* = 6. **(K–M)** Changes in protein abundances of collagen IV, α-SMA, vimentin, Wnt1, Wnt4, and β-catenin and miR-21 abundance in HK-2 cells. HK-2 cells were pretreated with 50 nM anti-miR21 or 50 nM anti-scramble 6 h prior to 48 h-AA treatment (2 μg/ml). *p < 0.05 and **p < 0.01 vs. anti-scramble controls, *n* = 3.

We further examined the potential connection between activation of miR-21 and Wnt/β-catenin signaling in a mouse model of AA-induced nephropathy (AAN). As shown in Figures 3A–D, protein levels of Wnt1, Wnt4, and β-catenin declined in the later stage (i.e., day 14 and 28) after anti-miR21 intervention, which was confirmed by immunohistochemistry (Figures 3H–J). Wnt2b and Wnt7a showed expression trends opposite to that of miR-21, and Wnt3 protein expression varied (Figures 3E–G), which suggested that miR-21 may have a direct negative effect on Wnt2b and Wnt7a.

Wnt2b is the only Wnt ligand predicted as a target of miR-21 by TargetScan v7.2 (http://www.targetscan.org/), but this remaines to be experimentally confirmed. A 3′-UTR luciferase reporter assay was performed to directly examine the 3′-UTR-mediated interaction between miR-21 and Wnt2b. The miR-21 mimic, compared to control, significantly reduced Wnt2b 3′-UTR activity by 95%, but have no significant effects on mutated Wnt2b 3′-UTR (Supplementary Figure S2A). The reciprocal repression relationship between miR-21 and Wnt2b in our AAN model further confirmed Wnt2b as a target of miR-21.

In line with the results of the *in-vivo* study, the protein levels of Wnt1, Wnt4, and β-catenin in HK-2 cells were attenuated following miR-21 inhibition. Meanwhile, protein abundances of α-SMA, collagen I, and collagen IV in HK2 cells were evidently decreased, suggesting that suppression of miR-21 could partially reverse the phenotypic changes induced by AA (Figures 3K,L).

### Absence of miR-21 Hinders AKI-to-CKD Transition by Selectively Downregulating Wnt/β-Catenin Signaling

**FIGURE 4:**
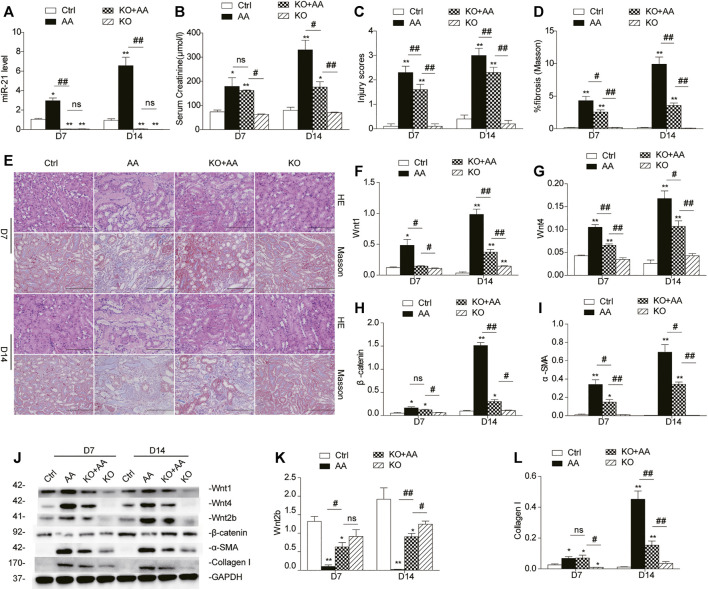
Absence of miR-21 hinders AKI-to-CKD progression by selectively downregulating the Wnt/β-catenin pathway. **(A)** Renal miR-21 abundance. The wild-type mice and miR-21 knockout mice were sacrificed on days 7 and 14 after AA administration. **(B)** Serum creatinine levels of mice. **(C)** Kidney injury scores of mice on days 7 and 14 post AA delivery. **(D)** Collagen abundance was analyzed using Masson’s trichrome staining. **(E)** Renal histologic patterns of wild-type mice and mir-21 KO mice (magnification, 200×, bar = 200 μm). **(F–L)** Relative protein expression of Wnt1, Wnt4, β-catenin, α-SMA, Wnt2b, and collagen I in kidneys. *p < 0.05, **p < 0.01 vs. controls. ^#^ *p* < 0.05, ^##^ *p* < 0.01 vs. KO + AA, *n* = 3.

To clarify the effect of miR-21 on Wnt/β-catenin signaling, we next studied the potential mechanism in miR-21^−/−^ mice. As shown in Supplementary Figure S2B, all our experimental mice were genetically identified as miR-21^−/−^ mice. Compared to wild-type mice, miR-21 knockout mice presented decreased serum creatinine levels on day 7 and 14 after AA administration (Figures 4A,B). Both H&E staining and Masson’s trichrome staining revealed a histological alleviation of kidney injury, with less renal tubular cast formation, tubulointerstitial fibrosis, and inflammatory cells infiltration in the miR-21^−/−^ mice (Figures 4C–E). Correspondingly, the upregulation of protein levels of wnt1, wnt4, and β-catenin, as well as of fibrotic indicators such as α-SMA and collagen I following AA exposure was attenuated in miR-21 KO mice (Figures 4F–L). Without AA exposure, there were no significant differences between the two groups in these parameters. Genetic deletion of microRNA-21 *in vivo* thus protected against AA-induced kidney injury and mitigated the detrimental profibrotic effects via downregulation of the canonical Wnt pathway.

### The Timing of Wnt/β-Catenin Inhibition Determines the Reversibility of AKI-to-CKD Transition in the AAN Mouse Model

**FIGURE 5:**
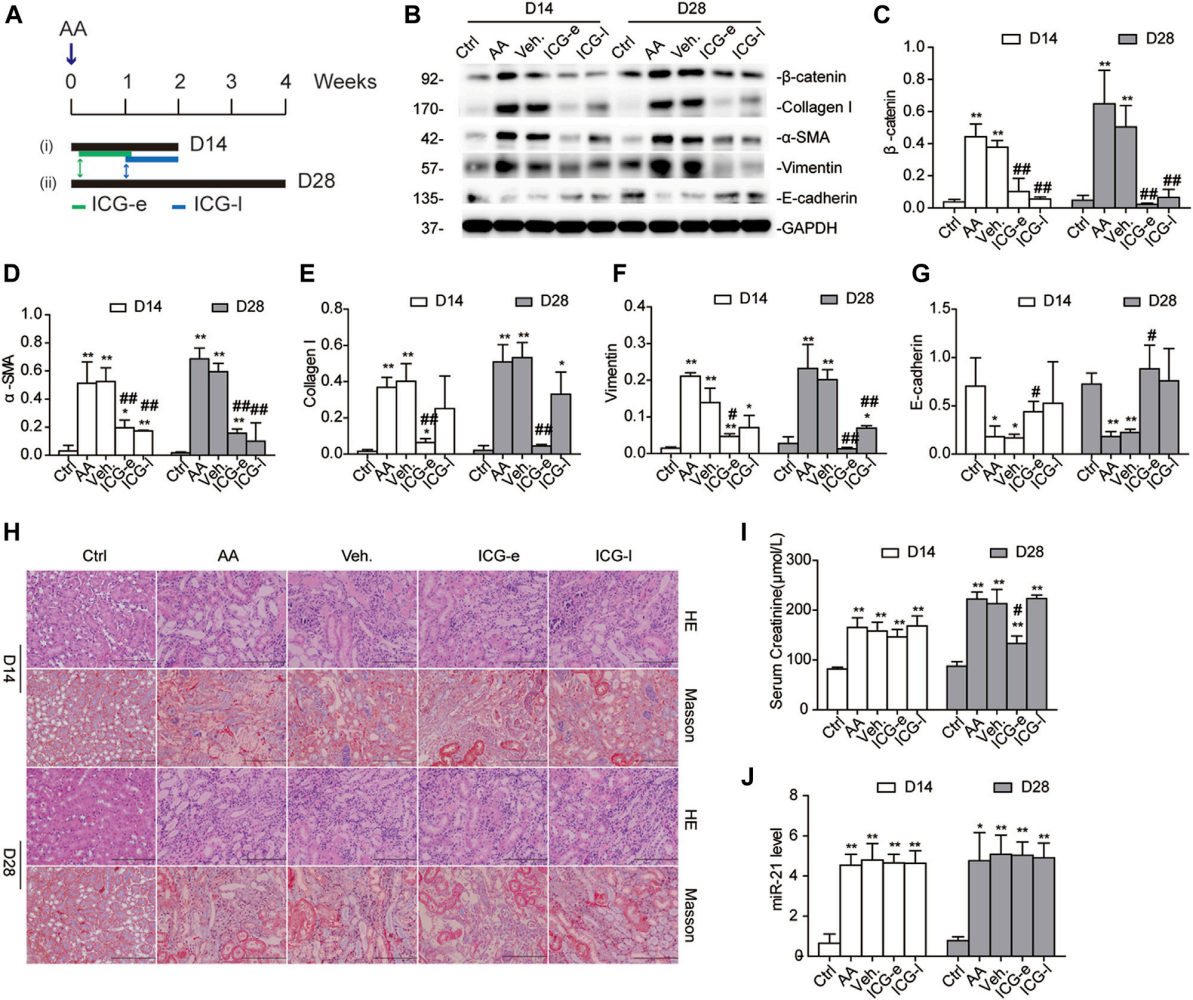
The timing of β-catenin inhibition determines the reversibility of AKI-to-CKD transition in the mouse AAN model. **(A)** Diagram showing the experimental design. ICG-001 (5 mg/kg) was administrated daily for consecutive 7 days starting 24 h after AA delivery (ICG-e) or 7 d after AA delivery (ICG-l). Veh, ICG-e and ICG-l refer to vehicle controls, early and late ICG-001 treatment, respectively. Mice were sacrificed 14 days and 28 days post AA administration. **(B–G)** Western blots and quantitative data of β-catenin, α-SMA, collagen I, vimentin, and E-cadherin. **(H)** H&E and Masson’s trichrome staining of the corticomedullary junction (magnification, 200×, bar = 200 μm). Tissue damage and renal fibrosis scores were calculated as described in the Methods. **(I)** Serum levels of creatinine on days 14 and 28 days after AA delivery. **(J)** RT-qPCR results of miR-21 abundance in the kidneys. *p < 0.05, **p < 0.01 vs. controls. ^#^ *p* < 0.05 and ^##^ *p* < 0.01 vs. vehicle controls (Veh), *n* = 3.

Considering the imperative role of β-catenin activation in driving AA-associated AKI-to-CKD transition, we speculated that targeted inhibition of this signaling may impede renal fibrogenic action followed by AKI. To this end, a small-molecule inhibitor (ICG-001) was applied to selectively inhibit gene transcription mediated by β-catenin (Eguchi et al., 2005; Henderson et al., 2010). ICG-001 (5 mg/kg) was administered daily for consecutive 7 days to mice, starting from 24 h after AA administration (early ICG-001 treatment group, ICG-e group), or starting from 7 days after AA administration (late ICG-001 treatment group, ICG-l group). The mice were sacrificed on day 14 or 28 after AA administration (Figure 5A). Interestingly, ICG-001 treatment markedly downregulated protein expression of β-catenin and α-SMA at each time point in both groups (Figures 5B–D). However, a decrease in serum creatinine was observed only in the ICG-e group on day 28 (Figure 5I), not on day 14, suggesting that timely inhibition of β-catenin was critical to effectively improve kidney recovery upon AA administration. Correspondingly, mice that received early intervention with ICG-001 showed less tubular damage and less interstitial fibrosis on day 28 than on day 14 (Figure 5H, Supplementary Figures S2C,D). As there was no significant difference in miR-21 abundance among the groups (Figure 5J), the Wnt/β-catenin pathway may have no reverse regulatory effect on miR-21.

To evaluate whether the antifibrotic effect of ICG-001 was independent of the severity of AKI, we observed dynamic changes in serum creatinine and renal histology on days 1, 3, and 7 after ICG-001 treatment. We observed no improvement in renal function or attenuation of renal fibrosis before day 7 (Supplementary Figures S2E–H). Thus, early treatment with ICG-001 protected against AA-induced renal fibrosis irrespective to the severity of AKI. Considering the serum creatinine levels, it seemed that early inhibition of β-catenin had limited effect on mouse kidney function in the acute phase post AA injury.

### Inhibition of Wnt1/4-Mediated β-Catenin Pathways Partially Reverses AA-Mediated Fibrogenesis in HK-2 Cells

**FIGURE 6:**
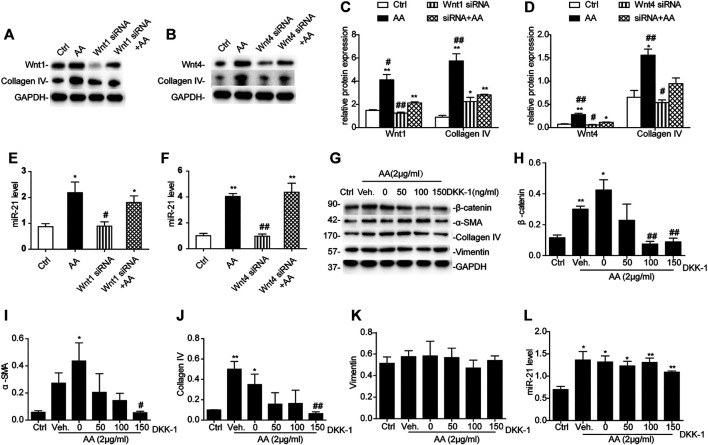
Inhibition of Wnt1/4-mediated β-catenin pathways partially reverses AA-mediated fibrogenesis in HK-2 cells. **(A–F)** Relative protein expression of Wnt1, Wnt4, and collagen IV and miR-21 expression in HK-2 cells treated with Wnt1 siRNA or Wnt4 siRNA 6 h prior to AA stimulation (2 μg/ml) for 48 h *p < 0.05 and **p < 0.01 vs. controls. ^#^ *p* < 0.05 and ^##^ *p* < 0.01 vs. Wnt1 siRNA + AA or Wnt4 siRNA + AA, *n* = 3. **(G–L)** RT-qPCR results of miR-21 abundance and changes in protein abundances of the indicated proteins in HK-2 cells treated with DKK-1 at 50, 100, or 150 ng/ml 1 h prior to AA stimulation (2 μg/ml) for 48 h *p < 0.05 and **p < 0.01 vs. controls. ^#^ *p* < 0.05 and ^##^ *p* < 0.01 vs. vehicle controls (Veh), *n* = 3.

Treatment with either Wnt1 siRNA or Wnt4 siRNA 6h prior to AA treatment significantly suppressed collagen IV protein expression (Figures 6A–D), but there was no significant change in the level of miR-21 (Figures 6E,F). When HK-2 cells were incubated with different concentrations of Dickkopf-related protein 1 (DKK-1), another potent β-catenin signaling blocker (Davidson et al., 2002; Mao et al., 2002), β-catenin activation secondary to AA exposure was remarkably hindered by DKK-1 at 150 ng/ml (Figures 6G,H). In addition, DKK-1 inhibited the upregulation of α-SMA and collagen IV protein expression following AA exposure (Figures 6I,J). The vimentin protein level and miR-21 abundance still remained unchanged after DKK-1 treatment (Figures 6K,L). These findings suggested that inhibition of β-catenin signaling by DKK-1 could suppress AA-mediated fibrogenic action in HK-2 cells.

### Overexpression of β-Catenin Augments Renal Fibrosis in AA-Induced AKI

To explore the role of the Wnt/β-catenin pathway in renal fibrosis further, mice were treated with LiCl (20 mg/kg/d), a β-catenin signaling activator (Narcisi et al., 2016), for seven consecutive days. The efficiency of LiCl in enhancing β-catenin abundance was verified by western blot analysis, both on day 14 and day 28 post AA treatment. The abundance of β-catenin was up-regulated much more on day 28 compared to that on day 14 (Supplementary Figures S3A,B). In comparison with untreated mice, renal expression of collagen I, α-SMA, and Snail1 was significantly increased in the LiCl-treated group, in line with the increase in serum creatinine (Supplementary Figures S3C–E). Further, pathological changes in the kidney tissue revealed that the overexpression of β-catenin concomitant with LiCl treatment exaggerated inflammatory cell infiltration, and promoted extracellular matrix deposition and the development of renal fibrosis (Supplementary Figures S3H–J). Of interest, miR-21 abundance in kidney tissue was not altered when β-catenin was activated (Supplementary Figure 3G). Given the consistent results on miR-21 *in vivo* and *in vitro*, we inferred that miR-21 might not be directly regulated by the Wnt/β-catenin pathway. In short, exogenous induction of β-catenin promoted renal fibrosis secondary to AA-induced AKI.

### LiCl Does Not Abolish the Anti-Fibrotic Effects Induced by Anti-miR21 Treatment

**FIGURE 7:**
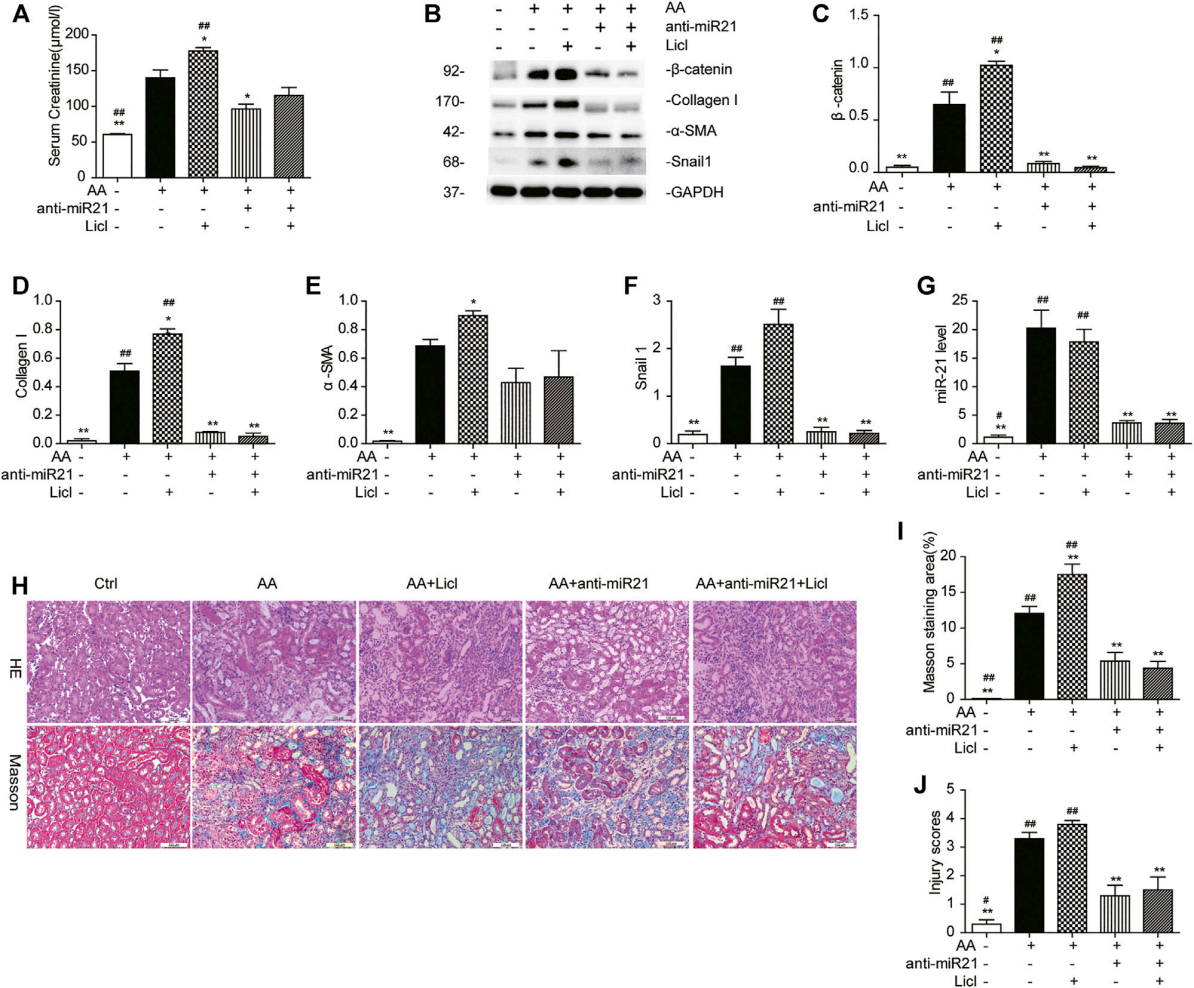
LiCl does not abolish the anti-fibrotic effects induced by anti-miR21 treatment. **(A)** Serum creatinine levels of mice with indicated treatment on days 14 after AA exposure. LiCl (20 mg/kg/d, 10 mg/ml) was administered 24 h post AA treatment, for 7 consecutive days. When LNA-anti-miR21 (10 mg/kg, 5 mg/ml) was applied, the oligos were administered via the tail vein within 60 min prior to AA delivery. **(B–F)** Western blots and quantitative data of β-catenin, collagen I, α-SMA and snail1 proteins. **(G)** RT-qPCR results for miR-21 in the kidneys. **(H–J)** H&E and Masson’s trichrome staining and analysis results (magnification, 200×, bar = 200 μm). *p < 0.05 and **p < 0.01 vs. AA. ^#^ *p* < 0.05 and ^##^ *p* < 0.01 vs. AA + Licl + anti-miR21 groups, *n* = 3.

To demonstrate the role of the canonical Wnt pathway in the pathogenesis of AA-induced AKI further, we treated mice with both anti-miR21 and LiCl. The combined use of AA and LiCl (20 mg/kg, i.p.) exacerbated renal injury (serum creatinine: AA vs. AA + LiCl, 140.2 ± 15.3 μmol/L vs. 175.4 ± 9.6 μmol/L, *p* < 0.05) and renal fibrosis (Figures 7A,H–J) on day 14 post AA treatment. At 20 mg/kg/d for 7 consecutive days, LiCl did not abolish the anti-fibrotic renoprotective effects bestowed by anti-miR21, as indicated by quantitative western blot data and RT-qPCR results for miR-21 abundance showed that overactivation of β-catenin by LiCl treatment was substantially suppressed upon anti-miR21 treatment (Figures 7B–G).

### Inhibition of miR-21/Wnt Signaling Mitigates Renal Inflammation and Oxidative Stress

**FIGURE 8:**
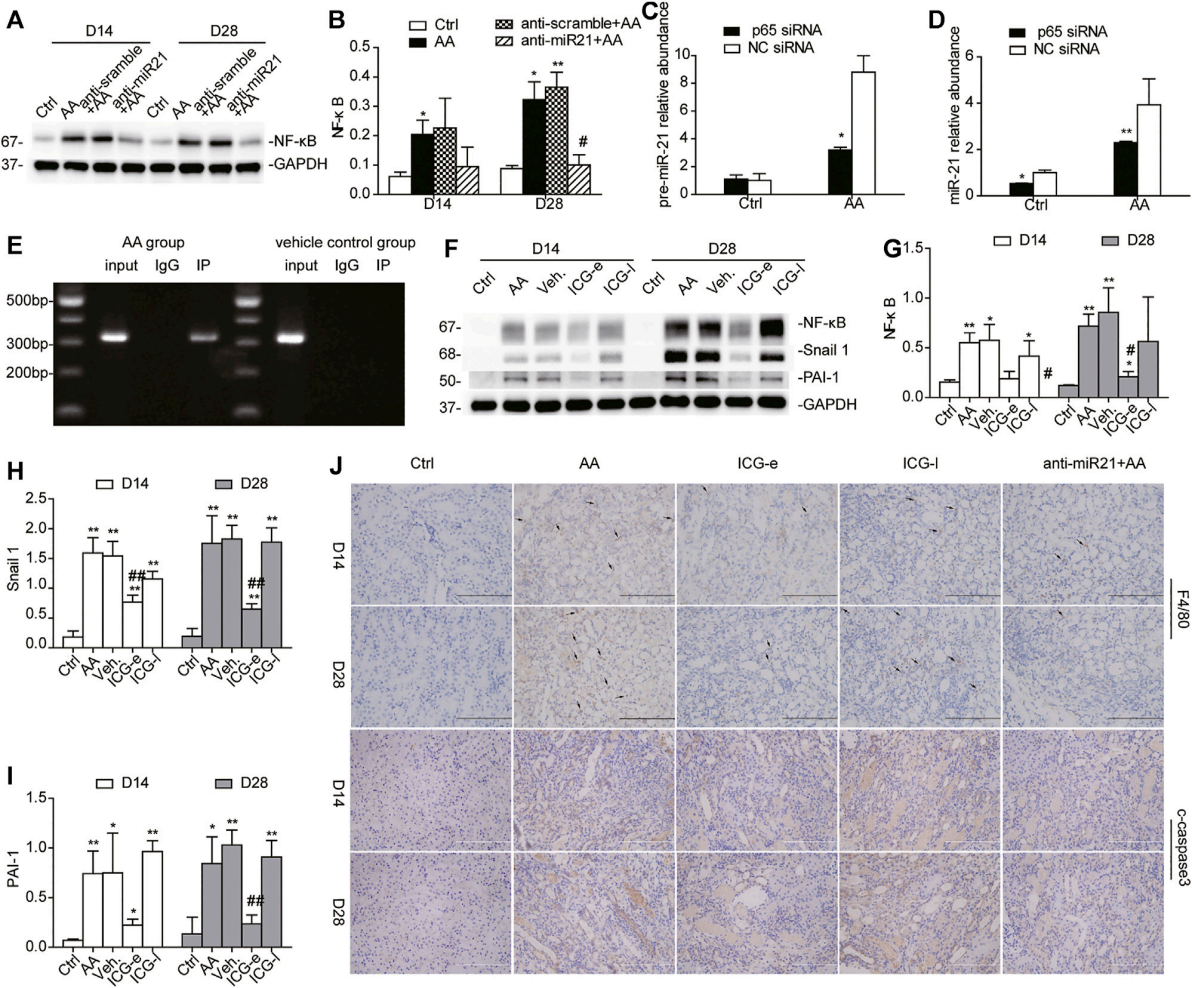
Inhibition of miR-21/Wnt signaling moderates renal inflammation and apoptosis. **(A, B)** Western blots and quantitative data of NF-κB protein in the kidneys. *p < 0.05 and **p < 0.01 vs. controls. ^#^ *p* < 0.05 and ^##^ *p* < 0.01 vs. anti-scramble controls. **(C,D)** Levels of pre-miR-21 and miR-21 in HK2 cells treated with NF-κB p65 siRNA 12 h before AA exposure. *p < 0.05 and **p < 0.01 vs. siRNA controls. **(E)** ChIP assay of NF-κB and miR-21 in mTECs in AA-treated and vehicle control groups. **(F–I)** Western blots and quantitative data of NF-κB p65, PAI-1, and snail1 in the kidneys. *p < 0.05 and **p < 0.01 vs. controls, ^#^ *p* < 0.05 and ^##^ *p* < 0.01 vs. vehicle controls (Veh), *n* = 3. **(J)** Immunohistochemistry staining of c-caspase three and F4/80 in the kidneys (magnification, 200×, bar = 200 μm). The black arrow indicates macrophage.

Immune inflammation, extracellular matrix deposition, and oxidative stress, which are triggered by AKI, are all involved in the development of CKD (Ko et al., 2008; Rodríguez-Iturbe and García García, 2010; Dounousi et al., 2012; Meng et al., 2014). We found that either inhibition of miR-21 or early blockage of β-catenin signaling downregulated the protein abundance of NF-κB, a key transcription factor responsible for inflammatory and immune responses (Figures 8A,B). On the other hand, treating HK2 cells with NF-kappa B p65 siRNA 12 h before AA exposure resulted in significant decreases in the levels of pre-miR-21 and miR-21 (Figures 8C,D). Bioinformatics analysis (PROMO database: alggen.lsi.upc.es/cgi-bin/promo_v3/promo/promoinit.cgi?dirDB=TF_8.3; TESS database: www.cbil.upenn.edu/tess) showed that the miR-21 promoter region has potential NF-κB binding sites located at approximately −3,000 bp upstream of the pre-miR21 5′-end. To verify the direct binding of NF-κB to the miR-21 promoter, we carried out a ChIP assay using mTECs. As expected, mTECs showed considerable NF-κB binding to the predicted regions of miR-21 (Figure 8E), suggesting that NF-κB was the transcription factor responsible for miR-21 transcriptional activation in HK2 cells. However, when mice were treated with ICG-001 at a late stage during the course of AKI, this beneficial effect disappeared (Figures 8F,G). Additionally, immunohistochemistry revealed that the cleaved (c-)caspase-3 positively stained area and the number of F4/80-positive cells were reduced after inhibition of miR-21/Wnt signaling, which was in accordance with the expression of NF-κB (Figure 8J, Supplementary Figures S4A–D). Similar results were found for plasminogen activator inhibitor-1 (PAI-1) and Snail1, two Wnt/β-catenin downstream profibrotic genes (Figures 8H,I) (Xiao et al., 2016) implying that delayed delivery of ICG-001 was incapable of hindering β-catenin-mediated gene transcription implicated in renal fibrogenesis. As shown in Supplementary Figure S4E, in the ICG-e groups, a battery of proinflammatory cytokines, including eotaxin, chemokine (C-X-C motif) ligand 1 (CXCl-1), and macrophage inflammatory protein-1β (MIP-1b), were markedly reduced when compared with the respective levels in the ICG-l and AA groups. Glutathione (GSH) levels and relative mRNA levels of manganese superoxide dismutase (*SOD2*), peroxisome proliferator-activated receptor γ (PPARγ) coactivator 1α (*PGC-1α*), and mitochondrial inner membrane protein (*Mpv17*) in the kidneys were all attenuated on day 14 and 28 after early ICG-001 delivery or inhibition of miR-21 (Supplementary Figures S4F–M). Taken together, the data indicated that miR-21 inhibition and early β-catenin blockade ameliorated apoptosis, macrophage infiltration, and inflammatory cytokine storm.

## Discussion

Our previous study demonstrated that in AAN model, excessive expression of miR-21 played a crucial role in driving AKI-to-CKD transition, which is an interest area of the current research. However, the key underlying mechanisms remains unclear and effective preventive or therapeutic approaches to impede CKD progression after AKI are still lacking. A recent study (Xiao et al., 2016) that reported that sustained Wnt/β-catenin signaling had a decisive role in mediating AKI-to-CKD progression in models of renal ischemia/reperfusion injury caught our attention. However, there was little direct evidence supporting the interaction between the two key pathways. This study provided novel data concerning the molecular mechanisms underlying the interaction between miR-21 and Wnt/β-catenin signaling in AA-induced AKI-to-CKD transition. We here report four main findings. First, our results demonstrated that chronic renal fibrosis after AA-induced AKI was related to a continuous activation of miR-21 and Wnt1/4-mediated β-catenin pathways. Second, we found that genetic ablation or pharmacologic inhibition of miR-21 markedly downregulated Wnt1 and Wnt4 canonical signaling, thus partially reversing renal tubulointerstitial fibrosis. Third, we provided evidence that the miR-21 level remained unchanged after inhibition or activation of Wnt/β-catenin signaling. Finally, our work suggested that only early use of a β-catenin inhibitor was beneficial to improve long-term outcomes of AKI by suppressing apoptosis, inflammation, and fibrosis.

An increasing number of studies have shown that abnormal Wnt/β-catenin activation is involved in renal fibrosis using several animal models (He et al., 2009; von Toerne et al., 2009; Hao et al., 2011; Maarouf et al., 2016; Xiao et al., 2016), for example, ischemia/reperfusion injury, unilateral ureteral obstruction (UUO), adriamycin-induced nephropathy, and renal transplantation. Among the 19 Wnt proteins that exist in mice, we focused on five representative Wnt ligands, i.e., Wnt1, Wnt2b, Wnt3, Wnt4, and Wnt7a. In previous studies (He et al., 2009; Xiao et al., 2016), these five proteins were strongly induced post ischemia/reperfusion injury, partially consistent with our observations that Wnt1 and Wnt4 displayed similar expression patterns *in vivo* and *in vitro*. However, Wnt2b, Wnt3, and Wnt7a decreased over time after AKI induction, especially in the later stage. Of all Wnt ligands, Wnt1 and Wnt4 are the most widely recognized to contribute to the pathogenesis of renal fibrosis. Wnt1 canonical signaling activates fibroblasts directly or via interaction with TGF-β, and therefore has become a topic of considerable importance related to fibrosis in various renal diseases (Young et al., 1998; Duan et al., 2012; Li et al., 2016; Maarouf et al., 2016). Wnt4/β-catenin was activated specifically in tubular epithelial cells and surrounding interstitial cells after kidney damage induced by UUO or folic acid (Surendran et al., 2002; Surendran et al., 2005; DiRocco et al., 2013). Possible explanations for these different findings in our and previous studies may include the use of different animal models, interactions among various Wnts, and differences in injury duration. Interestingly, β-catenin reached its highest level on day 28 after AA administration, lagging behind the peak levels of Wnt1 and Wnt4 on day 14 (Figure 1). These results suggested a synergistic effect of Wnts on β-catenin activation or other pathways independent of Wnt signaling. Further mechanisms remain to be discovered.

As described previously, miR-21 abundance as well as Wnt signaling play key roles in driving fibrotic responses post AKI. One aim of this study was to shed light on the relationship between miR-21 and the canonical Wnt pathway. To this end, experiments were performed using ICG-001 and LiCl as pharmacologic inhibitor and activator of β-catenin, respectively, and pharmacologic knockdown or genetic ablation of miR-21. We found that miR-21 deficiency or absence resulted in loss of Wnt1 and Wnt4 and induction of Wnt2b and Wnt7a, but had no effect on Wnt3. Apparently, in this animal model, miR-21 might serve either as a negative regulator or as a positive regulator of Wnt ligands. Since miRNAs mostly inhibit protein expression of their target genes through post-transcriptional repression (Ambros, 2004), the reciprocal suppression of miR-21 and Wnt2b identified in this study suggested that Wnt2b might be a target of miR-21. Whether the non-canonical Wnt pathway is involved in the progression of CKD remains unclear. However, a positive correlation between miR-21 and Wnt1/Wnt4-mediated β-catenin signaling was found *in vitro* (Wu et al., 2015). MiR-21 may regulate the Wnt pathway via directly targeting downstream genes, such as *DKK2* and *DDAH1* (Kawakita et al., 2014; Liu et al., 2016). In our study, miR-21 positively regulated the Wnt1/Wnt4 canonical pathway probably through upregulating genes upstream of Wnt. Further, we found that the Wnt/β-catenin pathway had no reverse regulatory effect on miR-21 both *in vivo* and *in vitro*. Taken together, our results indicated that miR-21 exerted versatile effects on the Wnt/β-catenin pathway. We are currently further investigating how miR-21 regulates Wnt/β-catenin signaling.

Another novel finding in this study was that the timing of inhibition of β-catenin signaling was pivotal for the reversal of post-AKI renal fibrosis. Compared to those in the ICG-l group, mice in the ICG-e had decreased β-catenin protein expression in the kidneys, less renal fibrosis, and lower protein levels of the β-catenin-downstream genes *PAI-I* and *Snail1*. Although protein expression of β-catenin and several fibrosis-related factors was downregulated in the ICG-l group, there was no obvious relief of renal tubulointerstitial fibrosis. Thus, in the current study, ICG-001 was effective only when applied early after damage induction. However, effective fibrosis alleviation even after late-stage administration has been reported in an UUO model and *in vitro* (Hao et al., 2011; Zhou et al., 2015). Thus, seeking an appropriate intervention timing seems crucial to improve the outcome of AKI.

Next, to investigate whether the reversal of AKI-to-CKD transition could be attributed to the inhibition of β-catenin signaling or was simply due to less severe injury, blood and kidney samples of AAN mice were harvest at different time points after AA administration. We found that ICG-001 blocked AKI-to-CKD transition irrespective of the severity of injury. Further, DKK-1-driven suppression of β-catenin reversed AA-induced phenotypic alterations. Given the pivotal role of the Wnt/β-catenin pathway in mediating AKI-to-CKD transition, we predicted that exogenous activation of β-catenin signaling through LiCl treatment would further aggravate renal tubular injury and promote extracellular matrix accumulation, which are characteristic features of advanced CKD. Indeed, LiCl treatment (20 mg/kg/d, for consecutive 7 days) did aggravate renal injury in the AAN mouse model. However, LiCl treatment did not rescue the downregulation of β-catenin and nor did it abolish the renoprotective effects bestowed by anti-miR21, suggesting additional β-catenin activation secondary to LiCl treatment might be far from offsetting the comprehensive effect bestowed by anti-miR21 treatment in our animal model.

The current study had several limitations that are worth mentioning. First, how miR-21 selectively regulated the different Wnts was not investigated. In this study, we confirmed that Wnt2b is a target gene of miR-21. However, it is not clear how miR-21 positively regulates the Wnt1/Wnt4-mediated canonical Wnt pathway. For example, it remains to be elucidated that how and which of the 19 Wnt ligands in vertebrates cooperate to promote AKI-to-CKD transition. Second, we established the *in-vivo* AAN model using a large dose of AA. Clinically, AAN is mostly caused by long-term exposure to low-dose AA. Further studies will be needed to examine whether the findings of the current study can be extrapolated to clinical application. Third, Wnt1/Wnt4 are mainly expressed in renal tubular epithelial cells and tubular interstitial cells, while we mainly used renal epithelial tubular cells in our *in-vitro* experiments. The complex interplay between TECs and other kidney intrinsic cells in establishing integrated outcomes might be worth in-depth investigation.

## Conclusion

In summary, sustained upregulation of miR-21 and selective activation of the Wnt/β-catenin pathways are responsible for excessive profibrotic gene expression, inflammatory cell infiltration, and increased cell apoptosis, thereby governing AKI-to-CKD progression. Targeted inhibition of either miR-21 or selective inhibition of Wnt/β-catenin signaling can relieve the transition to and progression of CKD, through restoring kidney function and attenuating renal fibrosis. Interestingly, ICG-001 exerted an antifibrotic effect only when given at the early stage after injury. We provide evidence that both miR-21 and the canonical Wnt pathway might be potential therapeutic targets for slowing down AKI-to-CKD progression.

## Data Availability

The raw data supporting the conclusions of this article will be made available by the authors, without undue reservation.

## Abbreviations

AKI, acute kidney injury; CKD, chronic kidney disease; AA, aristolochic acid; ESRD, end-stage renal disease; miRNAs/miRs, microRNAs; DMSO, dimethyl sulfoxide; DKK-1, dickkopf-related protein 1; NC, negative control; α-SMA, alpha-smooth muscle actin; PAI-1, plasminogen activator inhibitor-1; NF-κB, nuclear factor kappa-B; siRNAs, interfering RNAs; RT-qPCR, real-time quantitative PCR; SOD2, manganese superoxide dismutase; PPARγ, peroxisome proliferator-activated receptor superoxide γ; PGC-1α, PPARγ coactivator 1α; Mpv17, mitochondrial inner membrane protein; ChIP, chromatin immunoprecipitation; mTECs, mouse renal tubular epithelial cells; GSH, glutathione; EMT, epithelial-mesenchymal transition; AAN, AA-induced nephropathy; CXCl-1, chemokine (C-X-C motif) ligand 1; MCP-1, monocyte chemotactic protein 1; UUO, unilateral ureteral obstruction; TGF-β,transforming growth factor-β.
