# Supplementary figures and images for: Selective Wnt/β-Catenin Pathway Activation Concomitant With Sustained Overexpression of miR-21 is Responsible for Aristolochic Acid-Induced AKI-to-CKD Transition
Source: Front Pharmacol. 2021 May 28;12:667282. doi: 10.3389/fphar.2021.667282 (PMC8193720; doi:10.3389/fphar.2021.667282)

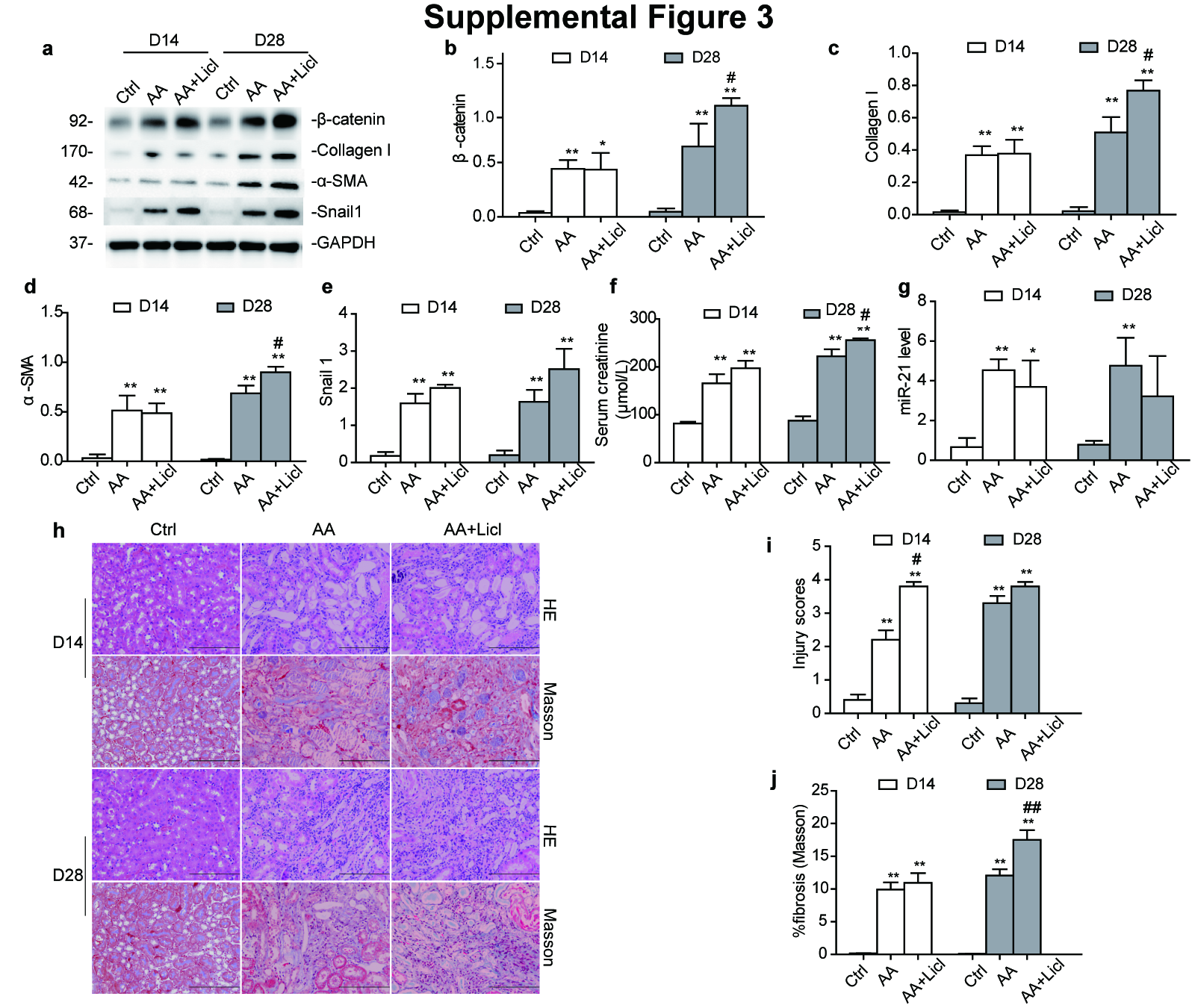

Supplement: Supplementary file 1 [file Image3.TIF]

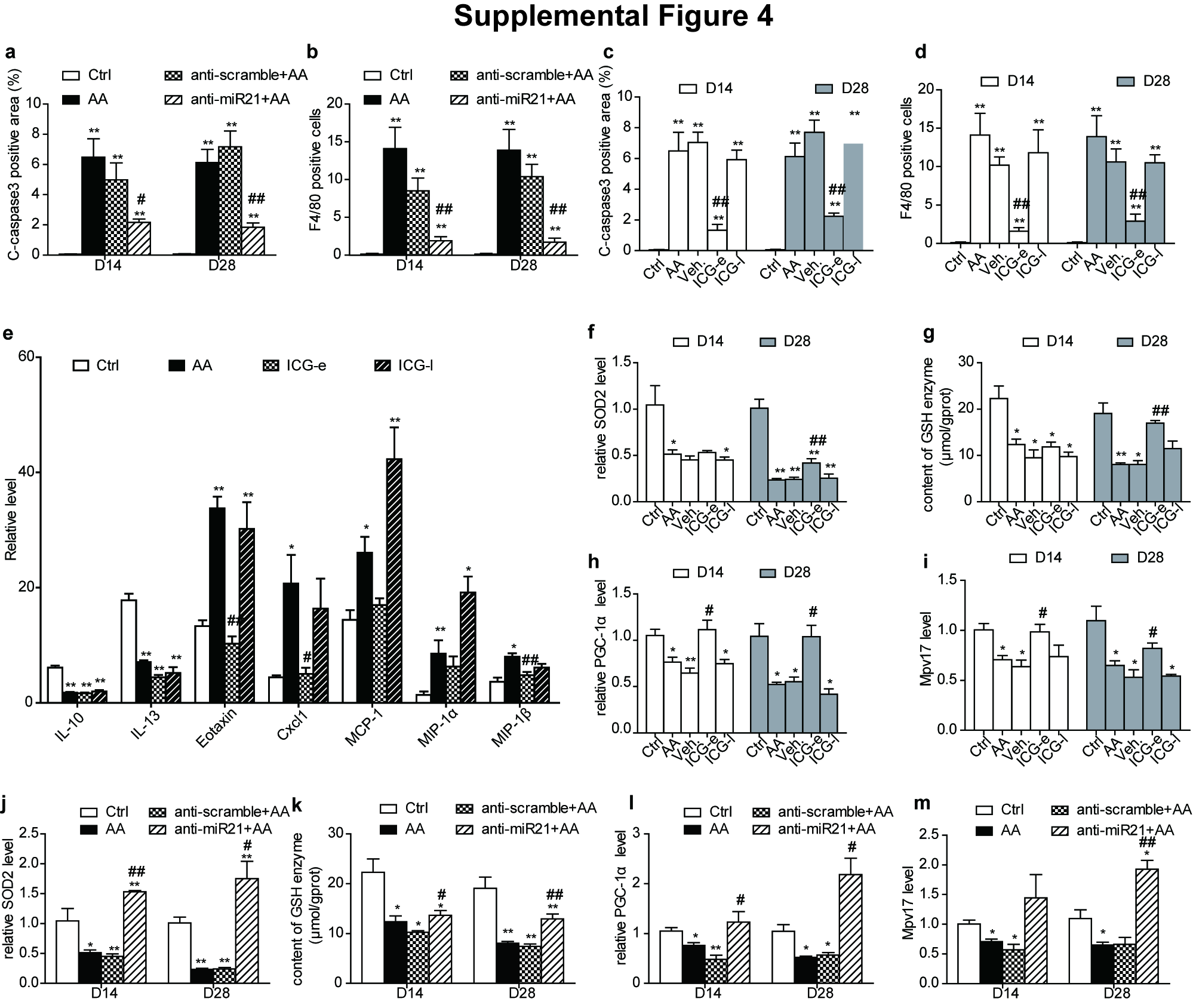

Supplement: Supplementary file 2 [file Image4.TIF]

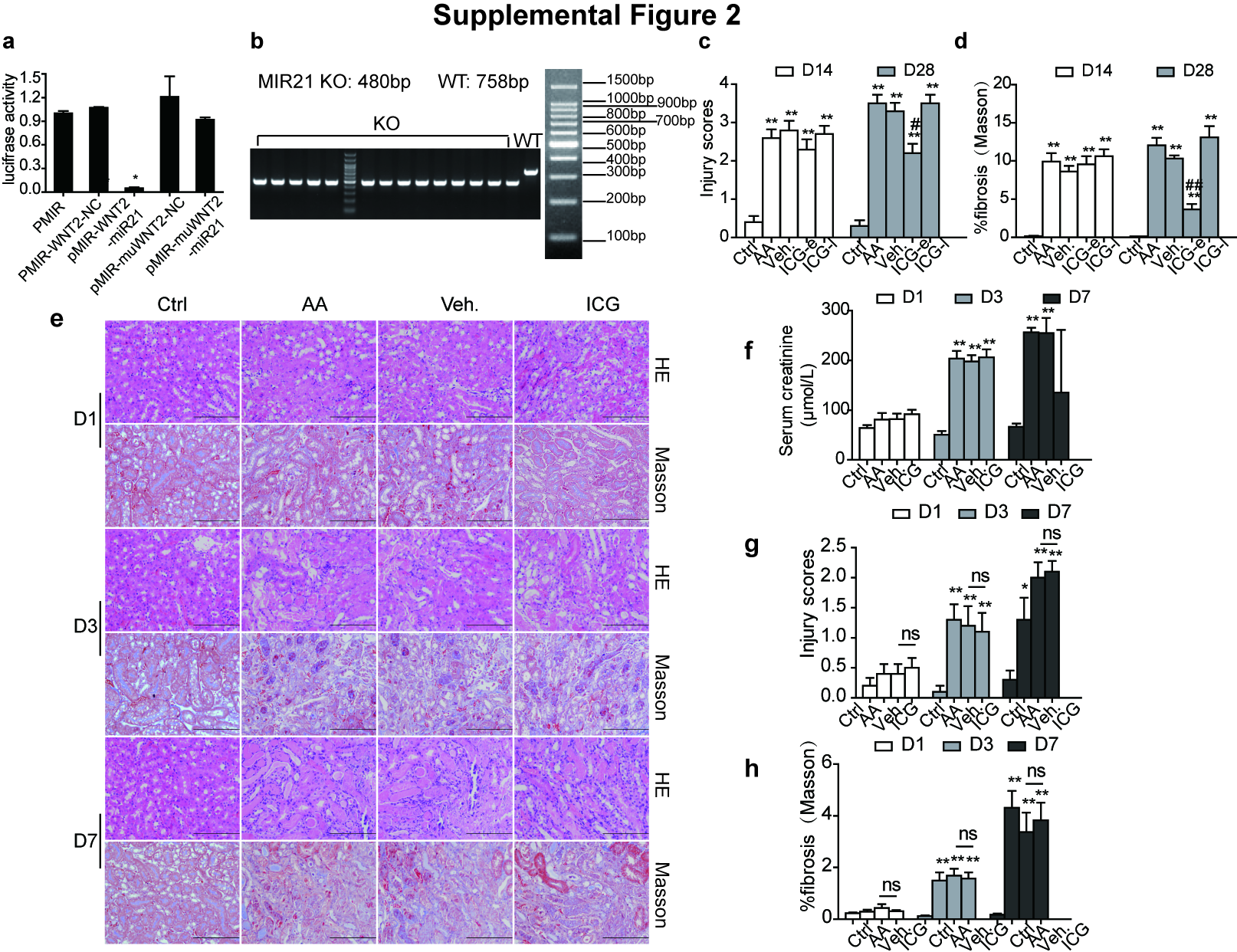

Supplement: Supplementary file 3 [file Image2.TIF]

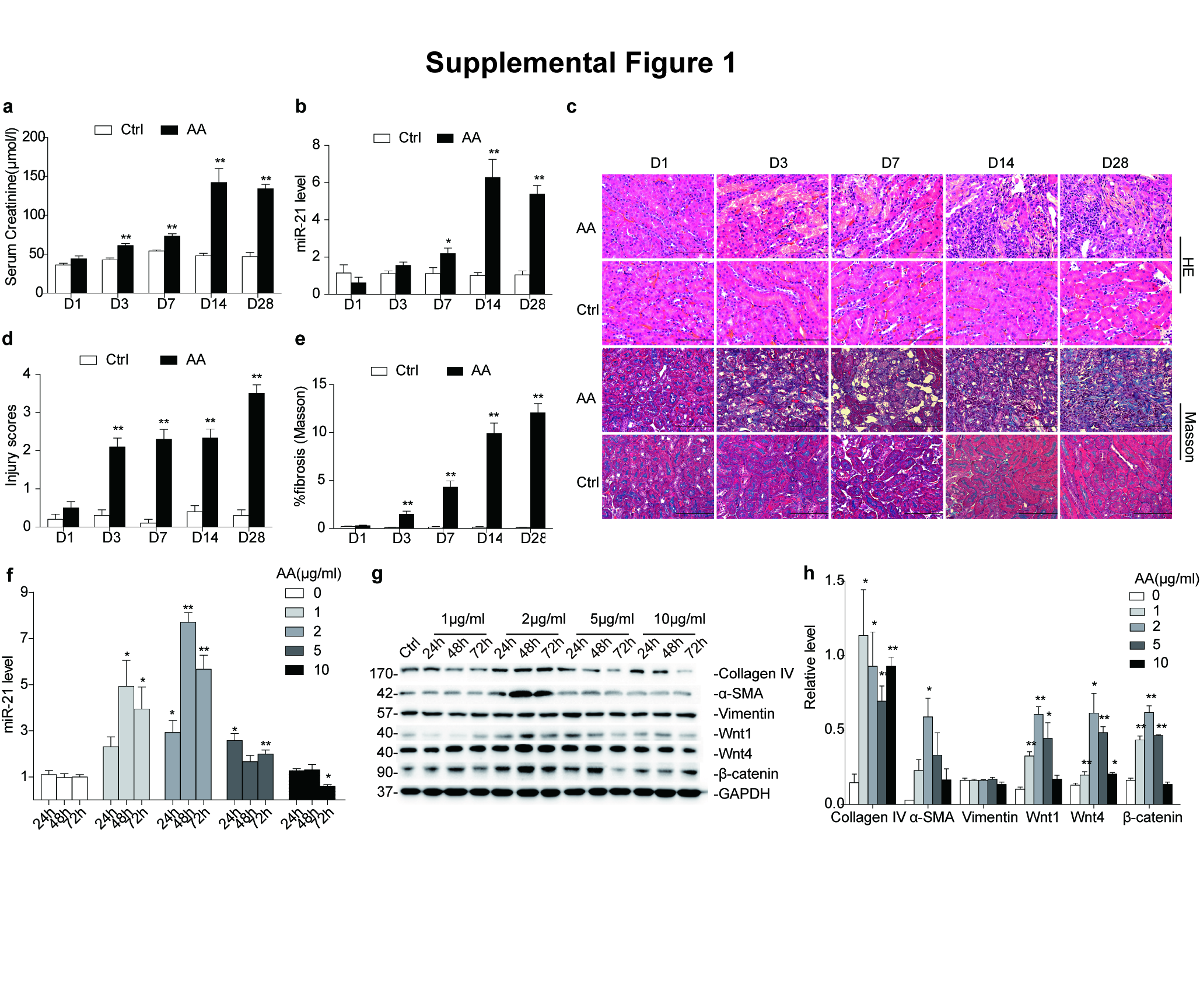

Supplement: Supplementary file 4 [file Image1.TIF]
